# Complications in Patients with Chronic Type B Aortic Dissection (cTBAD)—A Long-Term Analysis

**DOI:** 10.3390/life13030851

**Published:** 2023-03-22

**Authors:** Darya Mohajeri, Christos Rammos, Konstantinos Tsagakis, Thomas Schlosser, Arjang Ruhparwar, Tienush Rassaf, Rolf Alexander Jánosi, Julia Lortz

**Affiliations:** 1 Department of Cardiology and Vascular Medicine, West-German Heart and Vascular Center Essen, University of Duisburg-Essen, 45147 Essen, Germany; 2 Department of Thoracic and Cardiovascular Surgery, West-German Heart and Vascular Center Essen, University of Duisburg-Essen, 45122 Essen, Germany; 3 Department of Diagnostic and Interventional Radiology and Neuroradiology, University Hospital Essen, University of Duisburg-Essen, 45147 Essen, Germany

**Keywords:** aorta, aortic disease, aortic dissection, endovascular therapy

## Abstract

Chronic type B aortic dissection (cTBAD) is a rare but challenging condition that requires individual treatment strategies. Especially the long-term therapy impacts prognosis. In this single-center retrospective study, we evaluated patients with cTBAD in our vascular outpatient clinic over 10 years. Follow-up consultations included contrast-enhanced, electrocardiogram-triggered, high-resolution CT angiography (CTA) covering the entire aorta. Evaluated characteristics went beyond demographic characteristics combining the treatment approach and the timing and occurrence of potential complications. We analyzed 133 patients in total (n = 92, 69.2% male) with cTBAD with a mean follow-up of 67.7 months. Most of them underwent invasive treatment (n = 102, 76.7%), the majority received thoracic endovascular aortic repair (TEVAR) (n = 82, 61.7%). A total of 80 patients (60.2%) had major complications, whereas over a third was free of complications even after 5 years. Most common complications were progress of dissection and endoleaks, aneurysms of true (TL) and false lumen (FL) were more common in the later time periods. The treatment of cTBAD in terms of timing, therapy approach, and complications is still challenging for the entire aortic team. Nevertheless, the early recognition of complications permits promising treatment options and highlights the importance of frequent follow-up examinations especially within the first years.

## 1. Introduction

Chronic type B aortic dissection (cTBAD) is a rare but life-threatening disease of the aorta [1]. Symptoms are heterogenous, most frequently described are a sudden stabbing chest or back pain and malperfusional symptoms, e.g., a stroke, limb ischemia, or paraplegia [2]. First-line therapy of acute TBAD is thoracic endovascular aortic repair (TEVAR); however, for cTBAD, therapy and disease progression are more difficult because the majority of cTBAD patients require long-term monitoring and frequent therapy approaches for disease control [3,4,5]. This study aims to analyze the individual course of disease in order to find patterns for typical complications and to identify short- and long-term complications. The complication type and needs for reinterventions are investigated to optimize the aftercare and therapy of the disease. Previous observational studies regarding cTBAD have either evaluated smaller patient cohorts or observation periods were only a few years [6,7,8,9]. Aim of this study is to broaden the current knowledge of disease progress of cTBAD by assessing the different complications occurring over a long observation time. 

## 2. Materials and Methods

### 2.1. Study Design and Criteria

This is a retrospective observational study with an observation period of ten years. The study includes database research of all patients who presented in the Department of Cardiology and Vascular Medicine at the West German Heart and Vascular Centre of University Clinic Essen between 2009 and 2019. Inclusion criteria covered the existence of a chronic type B aortic dissection (cTBAD) diagnosed via computed tomography (CT) or magnetic resonance imaging (MRI) as well as clinical appearance. The admission was decided on according to the duration of the dissection, which was expected to be more than three months to be defined as a primarily chronic dissection. For patients with acute dissections (<2 weeks), inclusion only occurred if the dissection proved to have become chronic with a duration of over two weeks and thus be defined as a subacute dissection [10]. Exclusion criteria were further disease entities of an acute aortic syndrome without progression to a chronic type B dissection such as an intramural hematoma (IMH) or a penetrating aortic ulcer (PAU). An acute aortic dissection that did not develop chronically also led to exclusion. The patients’ age had to be over 18 years old as this study did not include pediatric patients. There were no exclusion criteria defined regarding gender.

### 2.2. CT Protocol

All patients underwent at least one CT angiography (CTA) which was performed contrast-enhanced, electrocardiogram-triggered, and with high-resolution (≤1.5 mm slice thickness). This was carried out with the latest CT generation, at the time of the data analysis in 2020, this ranged up to 384 slices dual source systems (SOMATOM Force, Siemens Healthineers, Forchheim, Germany). These scans were carried out continuously and ranged from the proximal supraaortic vessels down to the groin area. The CTA scans were analyzed with the Osiris software, version 5.5.2, 64 bit (Pixmeo Sàrl, Bernex, Switzerland). Our institution has a standardized examination protocol for CTA scans, inserted in the following: during the examination, the patients receive iodinated contrast (100–140 mL), which is infused at a rate of 4.0 mL/s via a peripheral vein (antecubital vein). For maximum concentration of the contrast in the aorta, a region of interest (RoI) is defined in the ascending part of the aorta. Data collection starts when the signal intensity in the RoI reaches a threshold of 120 Hounsfield units (HU). After 50 s, this process is repeated in the RoI in order to perform the venous CTA scan [11]. 

### 2.3. Follow-Ups

According to the current S2k guidelines of the AWMF (register number: 004-034) patients with cTBAD should be followed up at least once a year using CT or MRI [12]. After interventional treatment, the first check-up should take place during the hospital stay and another follow-up is recommended three months after being discharged from the hospital. Should there not be any serious complications during this time, further follow-up examinations are carried out at six-month intervals for the first two years and then annually [10]. The majority of this study’s patients were followed-up after three and six months and then presented with annual appointments. Some patients were followed up after 2 years since the original appointments were not kept by the patients.

### 2.4. Data Collection and Statistical Analysis

The data were collected from the medical archives of the Department of Cardiology and Vascular Medicine in the West German Heart and Vascular Centre of University Clinic Essen and were then documented in a Microsoft Excel 2019 database. The demographic data were taken via the hospital information system CGM Medico (© Compu Group Medical). After the complete documentation of all data in the Microsoft Excel 2019 database, these were evaluated anonymously. The data were analyzed using the IBM SPSS Statistics 27 program. By doing so, metric data were characterized by mean and standard deviation, whereas nominal values were evaluated by absolute and relative frequencies. Correlations of nominal data were assessed using the chi-square test and the phi test. A result was described as statistically significant with a value of *p* < 0.05.

## 3. Results

### 3.1. Patient Demographics and Therapeutic Approach

Overall, 133 patients were included, of which 92 (69.2%) were male. In average, patients were diagnosed with cTBAD at the age of 60.5 (±11.3) years, women approximately 5 years later than men. More than a third of all patients presented for five or more follow-ups. The majority of 102 patients (76.7%) received invasive therapy, of which 82 patients (61.7%) underwent TEVAR and 20 patients (15.0%) had to be treated surgically. From 2009 to 2019 only 31 patients (23.3%) were treated solely conservatively. We identified six vascular comorbidities and 18 other comorbidities that can be seen in Table 1 and Table 2. The most frequently occurring vascular comorbidity was an aneurysm of the ascending aorta, followed by aneurysms of the descending aorta. Other comorbidities covered cardiovascular diseases that are characteristic for aortic dissections, e.g., nearly all patients showed an arterial hypertension (99.2%). 

### 3.2. Complications

During the study, 80 patients (60.1%) developed major complications. Despite the long observation period, about 40% of patients remained free of complications even after more than five years. In order to give an overview of the time course of complications, we identified four different time periods: <30 days, 31 days to 1 year, 1 to 5 years and >5 years. This refers to no official classification of complications for cTBAD. Nevertheless, these time periods allow a more specific description for the development of complications. 

The majority of patients developed serious complications within the first five years after primary diagnosis. The highest rate of complications was found in the first year (n = 23, 17.3%) and the group of one to five years (n = 23, 17.3%), showing that these two time periods each include nearly a fifth of all patients with serious complications. Complications were defined as serious when leading to a change in therapeutic approach or necessitating a reintervention. Almost half of all serious complications occurred during the first year (47.5%). The most common early complications in interventionally treated patients presented as acute peri- and postinterventional complications, e.g., pericardial effusion in 13.3% of all early complications (n = 2) or acute ischemia in 20% of all patients during this period (n = 3). Within the first year, 15 patients needed stent extensions due to an acute progress of dissection or caused by an endoleak. In the group of 1 to 5 years as well as >5 years, aneurysms of the true lumen (TL) or the false lumen (FL) were the most common complications. Progress of dissection turned out to be the most common complication within the first five years. We defined this progress as the need for a postinterventional stent extension or clinical deterioration of the ischemia due to malperfusion. In the group of over five years, no progress of dissection could be documented. 

The occurrence of endoleaks was found in all four groups with its lowest rate in the first group of early complications (1 to 30 days). In total, 35 patients presented endoleaks (26.3%). However, not all cases of endoleak necessitated a reintervention and thus it was not always classified as a major complication. Most cases were an endoleak type 1 (n = 26, proportionally to all endoleaks ≙ 74.3%). An IMH or PAU was found in a total of four patients within the first five years, but none in the time period of over five years. The rarest complication appears as a long-term consequence of malperfusion, which occurs in only one case and results in shrinkage of the kidney with consecutive kidney failure. Another rare complication is a distal stent graft induced new entry (dSINE), which was found in two patients: one patient in the first year and one patient after more than five years. The risk of dSINE is increased if the stent graft is selected too large compared to the TL, resulting in distal oversizing (dOS) [13,14,15]. Cumulatively, 12.8% of all patients (n = 17) were admitted to the clinic as an emergency, of which about half of the admissions (n = 8) resulted from a covered rupture of the aorta. This is only about a fifth of all complications and thus shows that the majority of complications were not presented in an emergency setting, but in the regularly scheduled follow-ups. Pseudoaneurysms were found in 14 patients (10.6%), of which one-third (n = 5) developed within three months after the intervention and two-third (n = 9) occurred after a longer time period. They were mostly located in the descending aorta (n = 12). 

**Figure 1:**
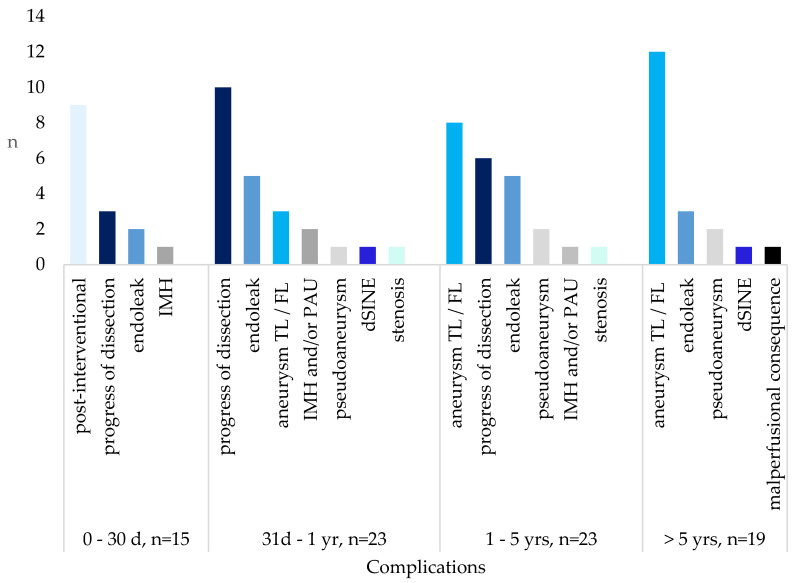
Frequency and type of complications in the different time periods. IMH = intramural hematoma, TL = true lumen, FL = false lumen, PAU = penetrating aortic ulcer, dSINE = distal stent graft induced new entry, d = day, yr = year.

An overview of all complications can be found in Table 3 and is illustrated in Figure 1. Many patients presented more than only one complication. We defined the reason for a reintervention as the main complication and thus the table shows just one complication per patient. Complications were not subdivided for men and women as there was no significant difference in the occurrence and the time course of both groups. Only for initial diagnosis we were able to establish a significantly lower age for men (59.0 ± 11.3 years) compared to women (64.0 ± 10.8). It clearly shows that the vast majority of all complications arose within the first five years after primary diagnosis. The lowest complication rate was found in the first period (1 to 30 days), thus indicating that acute postinterventional complications are not the most vulnerable phase of the disease.

### 3.3. Mortality

Although mortality was not a primary objective of the study, it showed that more than half of all patients (n = 69, 51.9%) were alive at the end of the study period. For 13 patients a follow-up regarding mortality was not possible. There was no significant correlation in neither the chi-square test nor the phi test between mortality and reintervention rate (X^2^(1) = 0.87, *p* = 0.352, φ = 0.87). From the 49 patients who underwent reintervention, 22 patients died, corresponding to a mortality rate of 44.9%, whereas of the 84 patients without reintervention 29 patients died, corresponding to a mortality rate of 34.5% (*p* = 0.352). In three reintervened patients and ten of the patients without reintervention mortality could not be evaluated. There was a statistically significant correlation between chronic kidney disease (CKD) and mortality (X^2^(1) = 4.46, *p* = 0.035, φ = 0.19), the presence of a descending aorta aneurysm and mortality (X^2^(1) = 5.97, *p* = 0.015, φ = 0.23) as well as endoleaks and mortality (X^2^(1) = 5.17, *p* = 0.023, φ = 0.21).

### 3.4. Aortic Remodeling

The majority of patients presented persistent perfusion in the false lumen (FL) despite therapy (n = 83, 62.4%). Among the conservatively treated patients 71% had persistent perfusion in the FL, whereas in the TEVAR patients the perfusion rates in the FL were significantly lower at 61%. This indicates aortic remodeling with successful elimination of the FL. In 25% of all patients (n = 34), the FL was thrombosed and blood flow was suspended, whereas 12% (n = 16) of all patients developed an aneurysm of the FL. The highest rate of FL aneurysms, 20%, was found in surgically treated patients. Patients after TEVAR developed FL aneurysms in only 12.2% of cases. Even among patients who received optimal medical therapy (OMT), the FL aneurysm rate was 6.5%, which can be explained by altered hemodynamics because of the persistent FL perfusion. It could be observed that patients after TEVAR presented higher rates of thrombosis of the FL, which also suggests positive aortic remodeling.

### 3.5. Reinterventions

**Figure 2:**
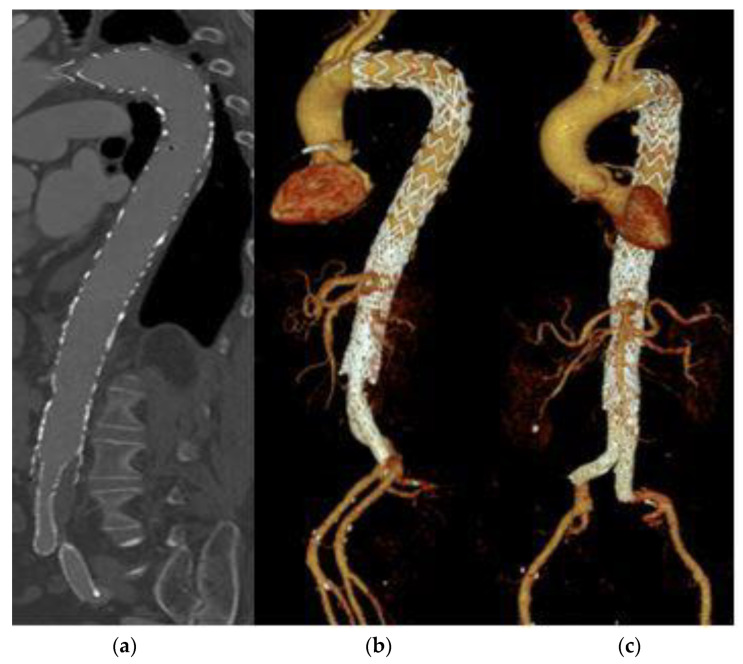
Aortic dissection after stent implantation and Y-prosthesis. (**a**) CT view from lateral, (**b**,**c**) show a 3D visualization of the entire aorta from lateral and ventral view respectively.

About a third of all patients (n = 49, 36.8%) required a reintervention. The reasons for a reintervention were various, mostly progress of dissection, an endoleak or the development of an aneurysm or pseudoaneurysm. The rarest cause for a re-evaluation of therapy was an insufficiency of sutures after the primary intervention (n = 1, 2.0%), which was only detected in one patient. In average, reintervention was performed after 27.5 months, but there was a wide range with a maximum time interval of 134 months until reintervention. About 15 patients (11.3%) had to undergo a second reintervention. This second reintervention was done after an average of 38 months. Five patients required three or more reinterventions. The maximum number of interventions was five reinterventions, this could be documented for one patient. Figure 2 shows an interventionally treated dissection of the aorta in three different views. On the left, the aorta is shown in a CT from the lateral side. In the middle and on the right, 3D images of the entire aorta are shown from the lateral and ventral views. In the area of the infrarenal aorta, an overlap of the aortic stent and the Y-prosthesis is evident. No endoleak is visible.

Using the chi-square test, a statistically significant connection could be established between the presence of endoleaks and reinterventions as well as pseudoaneurysms and reinterventions (*p* < 0.001 for both). It turned out that patients with a history of valve replacement had to undergo a reintervention significantly more often than patients with no history of any heart valve replacement (X^2^(1) = 8.25, *p* = 0.004, φ = 0.25).

## 4. Discussion

This study characterized 133 patients with cTBAD in an overall 10-year analysis. The results showed typical demographic data for cTBAD, the majority of patients were male with an age over 60 years. Characteristic cardiovascular comorbidities were present in nearly all patients, e.g., arterial hypertension, hypercholesterolemia, nicotine abuse, obesity, and arteriosclerosis [5].

Until now, several studies have examined the disease course of type B dissections. In 2019, an observational study that described 12 patients with cTBAD over two-and-a-half years was published. It was postulated that TEVAR serves as a sufficient therapy option for cTBAD patients and positive aortic remodeling rates could be found [6]. Because of the relatively short observation period and the smaller study group, the authors summarized that present results should be verified in larger observational studies and further discussed. Our results can verify these theses. We showed that based on a larger patient clientele with this specific disease, TEVAR remains the first choice of therapy for cTBAD. The decision on an endovascular therapy approach still depends on several factors [12]. 

Also in 2019, a retrospective study over ten years, in which all patients with a type B dissection (n = 32) were included, was finished [7]. Mortality served as a primary endpoint, while complications were not included in detail. The main focus was on factors that had an impact on mortality. They showed a positive correlation between chronic kidney disease (CKD) and mortality as well as further complications and mortality. In this regard, the study on hand is a valuable addition since the focus was set on the examination of complications and thus an important factor with an impact on mortality was discussed. In the analysis of the factors that affect mortality, we showed that CKD, an aneurysm of the descending aorta, and endoleaks have a statistically significant influence on increased mortality compared to patients in which these factors were not present. The results correspond to former studies but with the addition of the larger patient group [7]. 

Another observational study described subacute and chronic type B dissections undergoing endovascular therapy (n = 50) over 16 years [8]. Regarding aortic remodeling, the TEVAR patients showed similar rates as in this study. The authors hypothesized that the lowest survival rate was after about five years. This is also the time frame that was worked out to be crucial in terms of long-term observation within this study. We were able to present an overview of the complication rates and the characteristic time course of complications throughout the years after diagnosis of cTBAD in a large study group with different therapeutic strategies. 

According to an article on guidelines of type B aortic dissections from 2017, 25% to 40% of all TBAD have complicated courses. In the patient group at hand, nearly 60% presented with serious complications. However, the period of observation must be differentiated, as the complications documented here were followed up over several years after primary diagnosis, whereas other results may have mainly focused on acute and subacute complications [5]. It was claimed that approximately one-quarter of all patients with cTBAD require at least one reintervention [5]. Reinterventions are often necessitated by serious complications, making them a good comparative parameter with regard to the incidence of complications in cTBAD. In this study, approximately 36% of patients required at least one reintervention. The higher reintervention rate of our study compared with other studies that presented lower reintervention rates [16] may be due to several reasons: first, these study data were extracted from the archive of a specialized aortic outpatient clinic so it may be possible that compared with a hospital that is not specialized on aortic diseases, more complications tend to be diagnosed. Second, the strict adherence to frequented follow-ups may lead to earlier detection of complications, which can allow earlier reintervention and result in a better prognosis.

The reintervention rate showed no significant correlation with the mortality rate. Before, no survival benefit could be shown for patients without a reintervention [17,18], which can be confirmed by the data analyzed here. On average, the first reintervention occurred after 27.5 months, corresponding to approximately two years. For the second reintervention an even longer gap was documented, being carried out after an average of 38 months. This highlights the importance of long-term patient care, so that any complications can be recognized before manifesting clinical symptoms. 

Complications are more likely if the stent graft is not optimally adapted to the individual anatomical conditions. An inadequate fit of the stent graft increases the post-interventional risk of endoleaks, stent migration and progression of the dissection [19]. A stent graft that is chosen too large can lead to the development of a distal stent graft-induced new entry (dSINE) [20]. Optimal sizing of the stent graft to the anatomical conditions of the patient can be achieved by various imaging techniques. CT angiography is the imaging of choice. Intravascular ultrasound (IVUS) may provide a better imaging of the vessels [14]. In the existing clientele, IVUS was performed in one-third of patients, and stent graft planning was performed using accurate CT angiography fluoroscopy. Only two patients in the entire study developed a dSINE, indicating accurate pre-interventional diagnostics and peri-interventional optimization of stent graft selection.

In terms of therapy approach, TEVAR may be considered as first choice in uncomplicated TBAD with the presence of high-risk features, as this early intervention results in fewer long-term complications and a better outcome [12,21]. To determine the optimal timing of TEVAR, a study in 2020 investigated survival and complication rates of acute, subacute, and chronic dissections that received TEVAR (n = 314, time [t] = four years) [9]. Results showed that acute post-interventional complications are not the most vulnerable phase in cTBAD, but that the first years after TEVAR are crucial to prevent aneurysmatic changes as well as progressive dissections [9]. The results presented here confirm the study from 2020, that in cTBAD the first five years after primary diagnosis and after the first intervention are of utmost importance. Due to the exceptionally individualized disease course of cTBAD, many cases require further therapeutic steps [22]. This may be investigated in further studies based on this study’s results. In 2022, laser aortic septotomy was published as a new technology for optimizing cTBAD landing zones [23]. None of this study’s patients underwent this procedure but continued studies may show the advantage of this in a larger patient clientele as measures such as this can improve future TEVAR-based therapeutic pathways for cTBAD. 

Comparative studies regarding mortality after TEVAR or optimal medical therapy (OMT), a conservative therapy approach, showed no survival benefit after an observation period of two years, but the long-term results of the INSTEAD study showed a better survival rate for TEVAR compared to OMT after five years [21]. We could not establish a statistically significant difference in the survival rates of TEVAR versus OMT-treated patients. However, aortic remodeling was shown to be more successful in the TEVAR patients, which can be illustrated by the higher FL thrombosis rate of the TEVAR patients compared to the conservatively treated patients. Likewise, the positive aortic remodeling is shown by a 10% lower perfusion rate of the FL in the TEVAR patients (61%) compared with the conservatively treated patients (71%). Though aortic remodeling and thrombosis of the false lumen are crucial for positive long-term results, as proposed in a systematic review of 48 studies on endovascular cTBAD, many studies on this topic do not include quantifiable data on aortic remodeling [24]. Although not standardized, this difference in the perfusion rates in the FL of TEVAR patients compared to conservatively treated patients is very informative. Nevertheless, guidelines on standardization of quantification for aortic remodeling are needed in order to enable more precise comparisons. In 2022, a study with 41 patients that had received TEVAR in uncomplicated cTBAD was surveyed [25]. They showed no significant difference in the overall survival of patients with complete FL thrombosis compared to partial FL thrombosis. However, the group with complete FL thrombosis showed significantly lower rates of freedom of reintervention, which underlines the importance of FL lumen thrombosis regarding prognosis.

## 5. Conclusions

This study showed the variety of complicative events in the time course of cTBAD. TEVAR and OMT as well as measures of secondary prevention can give a stable therapeutic frame. Frequent follow-ups especially in the first five years after primary diagnosis or primary intervention help in identifying complications earlier so that these can be treated. An early reintervention can prevent the progression of complications and thus improve the patient’s prognosis.

## Figures and Tables

**Table 1:** Vascular comorbidities of the study group.

Comorbidity	n (%)
Aneurysm of the ascending aorta	45 (33.8)
Aneurysm of the descending aorta	29 (21.8)
Intramural hematoma (IMH)	21 (15.8)
Aneurysm of the infrarenal aorta	11 (8.3)
Penetrating aortic ulcer (PAU)	6 (4.5)
Aneurysm of the iliac artery	3 (2.3)

**Table 2:** Other comorbidities of the study group.

Comorbidity	n (%)
Arterial hypertension	132 (99.2)
Dyslipidemia, hypercholesterinemia	87 (65.4)
Nicotine abuse	60 (45.1)
Coronary heart disease	59 (44.4)
Aortic valve defect	58 (43.6)
Chronic kidney disease (CKD)	52 (39.1)
Obesity (BMI > 30 kg/m^2^)	41 (30.8)
PTCA and coronary stent implantation	28 (21.1)
Coronary bypass	17 (12.8)
Diabetes mellitus type 2	16 (12.0)
COPD (chronic obstructive lung disease)	16 (12.0)
Myocardial infarction	14 (10.5)
Valve replacement	12 (9.0)
Peripheral artery disease (PAD)	11 (8.3)
Apoplexy	10 (7.5)
Pacemaker	7 (5.3)
Connective tissue disease Marfan’s syndrome, Ehlers-Danlos syndrome, Loeys-Dietz syndrome	4 (3.0)
Bicuspid aortic valve	3 (2.3)

**Table 3:** Frequency and type of complications over time.

Time Frame	Complications	n (%)
0–30 days (n = 15)	Post-interventional	9 (60)
	Progress of dissection	3 (20)
	Endoleak	2 (13.3)
	Intramural hematoma (IMH)	1 (6.7)
31 days–1 year (n = 23)	Progress of dissection	10 (43.6)
	Endoelak	5 (21.7)
	Aneurysm of true (TL) or false lumen (FL)	3 (13.1)
	IMH and/or penetrating aortic ulcer (PAU)	2 (8.7)
	Pseudoaneurysm	1 (4.3)
	Distal stent graft-induced new entry (dSINE)	1 (4.3)
	Stenosis	1 (4.3)
1–5 years (n = 23)	Aneurysm of true (TL) or false lumen (FL)	8 (34.8)
	Progress of dissection	6 (26.2)
	Endoleak	5 (21.7)
	Pseudoaneurysm	2 (8.7)
	IMH and/or PAU	1 (4.3)
	Stenosis	1 (4.3)
>5 years (n = 19)	Aneurysm of true (TL) or false lumen (FL)	12 (63.2)
	Endoleak	3 (15.8)
	Pseudoaneurysm	2 (10.4)
	dSINE	1 (5.3)
	Long-term malperfusion (renal shrinkage)	1 (5.3)

## Data Availability

The data have been anonymized and can be sent per request.

## References

[1] Nienaber C.A., Clough R.E., Sakalihasan N., Suzuki T., Gibbs R., Mussa F., Jenkins M.P., Thompson M.M., Evangelista A., Yeh J.S.M. (2016). Aortic dissection. Nat. Rev. Dis. Prim..

[2] Parve S., Ziganshin B.A., Elefteriades J.A. (2017). Overview of the current knowledge on etiology, natural history and treatment of aortic dissection. J. Cardiovasc. Surg..

[3] Jánosi R.A., Rassaf T. (2020). Improving risk prediction in patients undergoing TEVAR for Type B Aortic dissection. Int. J. Cardiol..

[4] Kaji S. (2018). Update on the Therapeutic Strategy of Type B Aortic Dissection. J. Atheroscler. Thromb..

[5] Alfson D.B., Ham S.W. (2017). Type B Aortic Dissections: Current Guidelines for Treatment. Cardiol. Clin..

[6] Fujioka S., Irisawa Y., Horai T., Hosaka S. (2019). Endovascular Therapy for Chronic Type B Aortic Dissection. Ann. Vasc. Dis..

[7] Poleri I., Dias-Neto M., Rocha-Neves J., Sampaio S. (2019). Type B Aortic Dissection—A Single Center Series. Rev. Port. Cir. Cardio-Torac. Vasc. Orgao Of. Soc. Port. Cir. Cardio-Torac. Vasc..

[8] Hellgren T., Kuzniar M., Wanhainen A., Steuer J., Mani K. (2021). Clinical and Morphologic Outcomes of Endovascular Repair for Subacute and Chronic Type B Aortic Dissection. Ann. Vasc. Surg..

[9] Li D.-L., He Y.-J., Wang X.-H., He Y.Y., Wu Z.H., Zhu Q.Q., Shang T., Zhang H.K. (2020). Long-term Results of Thoracic Endovascular Aortic Repair for Type B Aortic Dissection and Risk Factors for Survival. J. Endovasc. Ther..

[10] Erbel R., Aboyans V., Boileau C., Bossone E., Di Bartolomeo R., Eggebrecht H., Vrints C.J. (2014). 2014 ESC Guidelines on the diagnosis and treatment of aortic diseases: Document covering acute and chronic aortic diseases of the thoracic and abdominal aorta of the adult. The Task Force for the Diagnosis and Treatment of Aortic Diseases of the European Society of Cardiology (ESC). Eur. Heart J..

[11] Lortz J., Papathanasiou M., Rammos C., Steinmetz M., Lind A., Tsagakis K., Schlosser T., Jakob H., Rassaf T., Jánosi R.A. (2019). High intimal flap mobility assessed by intravascular ultrasound is associated with better short-term results after TEVAR in chronic aortic dissection. Sci. Rep..

[12] Oberhuber A. (2022). S2k Leitlinie Typ B Aortendissektion.

[13] Lortz J., Leinburger F., Tsagakis K., Rammos C., Lind A., Schlosser T., Jakob H., Rassaf T., Jánosi R.A. (2019). Distal Stent Graft Induced New Entry: Risk Factors in Acute and Chronic Type B Aortic Dissections. Eur. J. Vasc. Endovasc. Surg..

[14] Lortz J., Tsagakis K., Rammos C., Horacek M., Schlosser T., Jakob H., Rassaf T., Jánosi R.A. (2018). Intravascular ultrasound assisted sizing in thoracic endovascular aortic repair improves aortic remodeling in Type B aortic dissection. PLoS ONE.

[15] Burdess A., Mani K., Tegler G., Wanhainen A. (2018). Stent-graft induced new entry tears after type B aortic dissection: How to treat and how to prevent?. J. Cardiovasc. Surg..

[16] Puech-Leao P., Estenssoro A.E.V., Wakassa T.B., Casella I.B., DeLuccia N. (2020). Long-term Results of Endovascular Treatment of Chronic Type B Aortic Dissection by Closure of the Primary Tear. Ann. Vasc. Surg..

[17] Arnaoutakis D.J., Khan T.A., Scali S.T., Neal D., Giles K.A., Cooper M.A., Beaver T.M., Huber T.S., Upchurch G.R., Arnaoutakis G.J. (2021). Remodeling, Reintervention, and Survival After Endovascular Repair of Chronic Type B Dissection. Ann. Thorac. Surg..

[18] Giles K.A., Beck A.W., Lala S., Patterson S., Back M., Fatima J., Arnaoutakis D.J., Arnaoutakis G.J., Beaver T.M., Berceli S.A. (2019). Implications of secondary aortic intervention after thoracic endovascular aortic repair for acute and chronic type B dissection. J. Vasc. Surg..

[19] Alexander S.A., Rubin G.D. (2009). Imaging the thoracic aorta: Anatomy, technical considerations, and trauma. Semin. Roentgenol..

[20] Jánosi R.A., Tsagakis K., Bettin M., Kahlert P., Horacek M., Al-Rashid F., Schlosser T., Jakob H., Eggebrecht H., Erbel R. (2015). Thoracic aortic aneurysm expansion due to late distal stent graft-induced new entry. Catheter. Cardiovasc. Interv..

[21] Nienaber C.A., Kische S., Rousseau H., Eggebrecht H., Rehders T.C., Kundt G., Glass A., Scheinert D., Czerny M., Kleinfeldt T. (2013). Endovascular repair of type B aortic dissection: Long-term results of the randomized investigation of stent grafts in aortic dissection trial. Circ. Cardiovasc. Interv..

[22] Loskutov A., Cooley M., Scheidt M., Mansukhani N., Hart J., Hieb R.A., Rossi P.J., Patel P.J. (2021). Endovascular Management of Chronic Type B Aortic Dissection. Tech. Vasc. Interv. Radiol..

[23] Fukuhara S., Tchouta L., Pampati R., Liesman D.R., Khaja M.S. (2022). Laser aortic septotomy during thoracic endovascular aortic repair for chronic type B aortic dissection. J. Thorac. Cardiovasc. Surg..

[24] Williams M.L., de Boer M., Hwang B., Wilson B., Brookes J., McNamara N., Tian D.H., Shiraev T., Preventza O. (2022). Thoracic endovascular repair of chronic type B aortic dissection: A systematic review. Ann. Cardiothorac. Surg..

[25] Kong M., Ni M., Zhu X., Qian J., Duan Q., Song J., Feng Z., Dong A. (2022). False lumen patency status and outcomes after endovascular repair of uncomplicated chronic type B dissection. Catheter. Cardiovasc. Interv..

